# Optimising and Communicating Options for the Control of Invasive Plant Disease When There Is Epidemiological Uncertainty

**DOI:** 10.1371/journal.pcbi.1004211

**Published:** 2015-04-13

**Authors:** Nik J. Cunniffe, Richard O. J. H. Stutt, R. Erik DeSimone, Tim R. Gottwald, Christopher A. Gilligan

**Affiliations:** 1 Department of Plant Sciences, University of Cambridge, Cambridge, United Kingdom; 2 United States Department of Agriculture, Agricultural Research Service, Fort Pierce, Florida, United States of America; University of New South Wales, AUSTRALIA

## Abstract

Although local eradication is routinely attempted following introduction of disease into a new region, failure is commonplace. Epidemiological principles governing the design of successful control are not well-understood. We analyse factors underlying the effectiveness of reactive eradication of localised outbreaks of invading plant disease, using citrus canker in Florida as a case study, although our results are largely generic, and apply to other plant pathogens (as we show via our second case study, citrus greening). We demonstrate how to optimise control via removal of hosts surrounding detected infection (i.e. localised culling) using a spatially-explicit, stochastic epidemiological model. We show how to define optimal culling strategies that take account of stochasticity in disease spread, and how the effectiveness of disease control depends on epidemiological parameters determining pathogen infectivity, symptom emergence and spread, the initial level of infection, and the logistics and implementation of detection and control. We also consider how optimal culling strategies are conditioned on the levels of risk acceptance/aversion of decision makers, and show how to extend the analyses to account for potential larger-scale impacts of a small-scale outbreak. Control of local outbreaks by culling can be very effective, particularly when started quickly, but the optimum strategy and its performance are strongly dependent on epidemiological parameters (particularly those controlling dispersal and the extent of any cryptic infection, i.e. infectious hosts prior to symptoms), the logistics of detection and control, and the level of local and global risk that is deemed to be acceptable. A version of the model we developed to illustrate our methodology and results to an audience of stakeholders, including policy makers, regulators and growers, is available online as an interactive, user-friendly interface at http://www.webidemics.com/. This version of our model allows the complex epidemiological principles that underlie our results to be communicated to a non-specialist audience.

## Author Summary

Increases in global trade and travel suggest outbreaks of plant disease caused by invasive pathogens will increase in frequency. We use mathematical modelling to show how control of such disease outbreaks can be optimised. Although our methods and analyses are generic, we use the attempted eradication of citrus canker from Florida (1996–2006) as a case study, and focus upon the performance of reactive culling (i.e. removal of all host plants within a certain distance of detected infection). We show how the cull radius can be optimised, even when there is significant cryptic infection (i.e. infection without visible symptoms). The inherent randomness of disease transmission implies a control strategy can lead to a number of outcomes: the optimal strategy therefore depends on the level of risk that is tolerable. We also consider balancing local vs. global impacts of disease. We show how it can be optimal to control initial outbreaks very extensively, even though this would lead to many local removals, since timely local eradication reduces the risk of a devastating large-scale epidemic. Our model is available as an interactive, user-friendly interface at http://www.webidemics.com/, intended to illustrate the sometimes counter-intuitive epidemiological principles that underlie successful disease control.

## Introduction

Impacts of invading pathogens can be extremely severe, and so understanding how controls can be optimised is imperative [1]. We focus here on plant disease, motivated by the serious and potentially irreparable ecological damage that can follow introductions of plant pathogens into natural host populations [2], and the obvious food security and economic implications of epidemics in crops [3–5]. Increased global trade and travel mean the risk of introduction of exotic pathogens can only reasonably be expected to increase [6], which in turn indicates control of invasive plant disease is likely to remain important for many years to come.

We target optimising reactive eradication of small-scale outbreaks of an invading plant pathogen [7–10] occurring in regions extending from 1-10km. We concentrate on how cryptic infection (i.e. infectivity without symptoms), the inherent stochasticity of epidemics, and uncertainties in the parameters controlling disease spread affect the performance of control via local removal of plant hosts in the vicinity of detected infection. Effectively controlling in this fashion is extremely challenging, requiring an estimate of how far the epidemic has spread ahead of visibly infected regions. Nevertheless, recent modelling studies of a number of plant pathogens have shown how, in principle, control by local removal of susceptible hosts can be effective in managing plant disease [11–13], even when disease spreads non-locally via complex contact networks [14–18]. A consensus has begun to emerge that this type of control can be successful, albeit with the beguilingly simple proviso that there is a need to “match the scale of control with the intrinsic scale of the epidemic” [19]. The obvious problem is that the appropriate scale is very difficult to define, and depends in a complex fashion on the interplay between the epidemiology of the plant-pathogen interaction, the spatial distribution of susceptible hosts, the implementation and logistics of detection and control, and the current state of the epidemic. The quantitative detail of how these factors affect the nature of the optimal control strategy and how effectively it performs is extremely complex, and general principles remain ill-understood. However, identifying such general principles is clearly relevant to the control of all plant diseases.

Here we use mathematical modelling to investigate epidemiological principles underlying successful control. We consider a range of strategies for management of a newly invading plant pathogen, and identify optimal control scenarios that minimise the “epidemic impact”; we define this to be the total of both the number of hosts lost to disease and healthy hosts removed by control. We show the importance of allowing for the inherent stochasticity of epidemics in comparing control scenarios by analysing the empirical distribution of epidemic impacts for fixed values of epidemiological and control parameters. In particular, we show how the optimal control strategy changes when we take account of different levels of risk aversion [20]. We further analyse the effects of critical epidemiological and logistic factors on disease control. The following are considered: the rate and spatial scale of disease spread, the initial level of infection, the ability to detect disease, the length of time infection remains cryptic, the frequency of surveying and any notice period or other delay before removal. We also show how accounting for the risk of export of inoculum outside of the region of immediate interest leads to optimum control strategies that differ from those derived by focusing solely on local impacts [21,22]. Finally we consider how control is adversely affected if pathogen spread occurs via a thick-tailed dispersal kernel, although we demonstrate that even then an optimal control strategy can still be defined.

Although the issues we investigate are generic, we illustrate our work in the context of an economically important system for which eradication by reactive culling was attempted: citrus canker (caused by the bacterium *Xanthomonas citri* pv. *citri*) on commercial and residential citrus in Florida. The United States government spent over one billion dollars on survey, control and compensation costs during an eradication campaign that ran from 1996 to 2006 [9] (see S1 Text for further details). Aside from the attraction of choosing a prominent and controversial real-world example to frame our analyses, a major motivation for using this system as our case-study is an extremely detailed data set on disease spread in five uncontrolled sites in the Miami region, originally collected by the United States Department of Agriculture [23]. These data have allowed parsimonious, stochastic, spatially-explicit, epidemiological models to be fitted to the spread of citrus canker that track the disease status of individual host plants [11,12,24,25], and here we use a flexible extension of these models to analyse the effectiveness of the control scenarios we consider. We also take advantage of recent work fitting this type of model to huanglongbing disease (also known as HLB or citrus greening) [26], a disease of citrus vectored by psyllids and that is caused by *Candidatus* Liberibacter spp. bacteria, in order to demonstrate the flexibility of our methods and the generality of the underlying principles.

A principal challenge in using epidemiological models to inform policy is rendering the assumptions and outputs of models in forms that can be readily understood and interrogated by policy makers [27]. What is needed is a tool to allow epidemiological and disease control scenarios to be explored by regulatory decision makers. This would not only help ensure appropriate action is taken, but would also help ensure factors affecting the success (and the risk of failure) of a preferred control strategy are understood by those who have to make and justify decisions. Epidemiologists have typically adopted a “black-box” approach, in which the analytical process is hidden and where only the resulting control recommendations are delivered. This lack of transparency makes it difficult and in some cases impossible for stakeholders affected by control to question the scientific basis of decision making, leading to controversy and even to less effective control [28]. Accordingly we introduce a user-friendly interface to our model, enabling the effects on control performance of changes to disease spread and control parameters to be explored, which as we demonstrate here can include applying the model to different pathosystems. By allowing for an ensemble of simulation runs with identical parameters, this ‘front-end’ also allows stochastic variability to be visualised. The front-end is available online at http://www.webidemics.com/ (Webidemics is a backronym: (WEB)-based (I)nteractive (D)emonstration of (E)pidemiological (M)odelling (I)nforming (C)ontrol (S)trategies). The Webidemics interface demonstrates the challenges inherent in optimising control strategies that account for cryptic infection, stochasticity and uncertainty in parameter values.

## Methods

### Host landscape

Our model readily accommodates an arbitrarily complex host landscape, from regular geometrical patterns typical of agricultural and horticultural crops, to random and clustered patterns typical of plant hosts in natural environments. With that generality in mind, we illustrate the approach for a random host landscape typical of urban Florida, with 2000 trees randomly distributed on a 2km x 2km square at a plausible density for dooryard citrus (i.e. trees in residential gardens) [12,25]. We note our Webidemics interface also accommodates a citrus grove (the colloquial term for what is also referred to as a “citrus orchard”), with 2016 hosts in two adjacent blocks, planted in rows 10m apart and with a 5m within-row host spacing, reflecting standard practice in the U.S. citrus industry. For brevity all results presented in this paper for citrus canker correspond to the random host landscape (note, however, that our application of the model to HLB considers disease spread through the citrus grove host landscape).

### Epidemiological model

**Fig 1:**
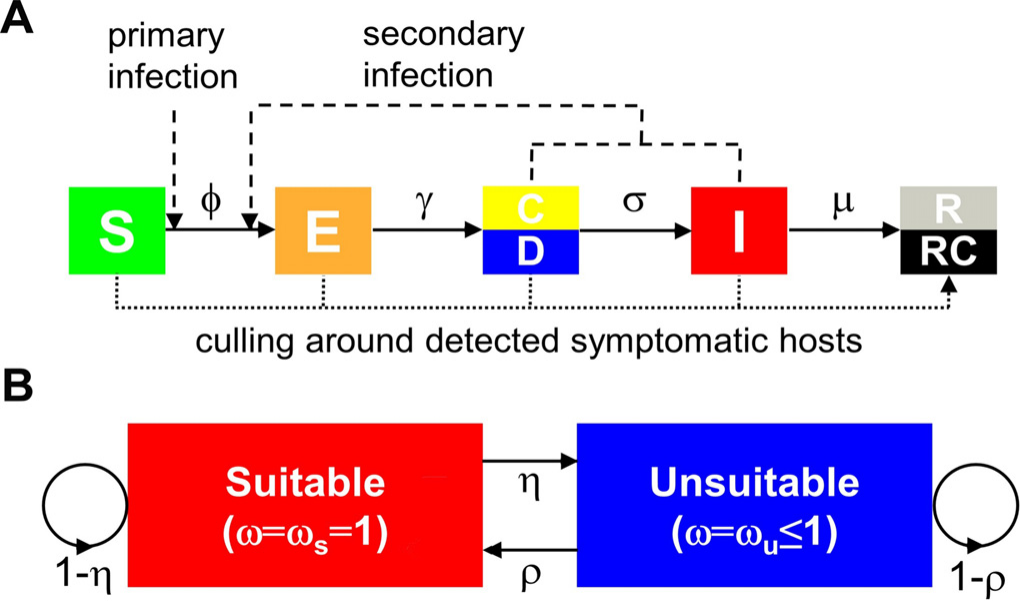
The epidemiological model. (a) Transitions between compartments: (S)usceptible, (E)xposed, (C)ryptic or (D)etectable, (I)nfected and (R)emoved. (b) Markov chain model for environmental conditions; a transition occurs every *T*_*w*_ units of time.

We use a spatially-explicit, stochastic, individual-based, compartmental model to represent the spread of a plant pathogen through a population of *N* hosts (Fig 1a). Hosts are categorised by disease status: (S)usceptible hosts are uninfected; (E)xposed hosts are latently infected, and so are neither symptomatic nor infectious; (C)ryptic hosts are infectious but still asymptomatic; (D)etectable hosts are symptomatic but not infectious; (I)nfected hosts are both infectious and symptomatic; and (R)emoved hosts are epidemiologically inert, either because of disease-induced death or because the host has been removed by any control effort. The Webidemics interface allows the timing of the cryptic and detectable classes to be exchanged, with visible symptoms either preceding or following the onset of infectiousness, and therefore can represent any of the SECI, SEDI, SECIR and SEDIR epidemic models [19]. Here, motivated by the biology of citrus canker [24,29,30], we concentrate exclusively on the SECI and SECIR variants of the model, in which detectable symptoms strictly follow infectiousness. This is the case in which control is most difficult, since it is hampered by invisible cryptic infection. We note that, although citrus canker does not itself directly kill host plants, accounting for control requires there to be a removed compartment in the model, as does allowing the user of our Webidemics interface to apply the model to host-pathosystems for which there is disease-induced host death.

In the absence of control, the E to I and C/D to I transitions occur at fixed rates γ and σ, and so waiting times in these compartments are exponentially distributed, with means 1/γ and 1/σ, respectively (see Table 1). This assumption could of course readily be relaxed to allow for other distributions of waiting times [31]. In the SECIR and SEDIR variants of the model, the rate of disease-induced death is μ, again with an exponentially distributed transition time (mean 1/μ). For any given host plant, the rate of the S to E (susceptible to exposed) transition depends on the status of other hosts and the suitability of the environment for infection. In particular, if host *i* is susceptible at time *t* then it becomes latently infected (i.e. transitions to the E compartment) at rate

$$\phi_{i}(t)=\omega (t)\left(\varepsilon +\beta \sum_{j}K(d_{ji};\alpha )\right),$$ (1)

where the summation runs over all infectious hosts, *j*, and where host *j* is at distance *d*_*ji*_ from host *i*. The underlying maximal rates of primary and secondary infection are ε and β, respectively, and *ω*(*t*)≤1 parameterises any time-variation in environmental suitability for infection (see below).

**Table 1:** Definition of symbols and default values of parameters (based on values from [11,12,24,25,29,30]).

	Description	Default Value
*N*	Total number of hosts	2000
γ	Rate of onset of infectiousness (average latent period 1/*γ*)	0.1d^-1^
σ	Rate of emergence of symptoms (average incubation period 1/*γ*+1/*σ*)	0.01d^-1^
μ	Rate of disease-induced death (average infectious period 1/*σ*+1/*μ* if μ > 0, otherwise infinite)	0 d^-1^(i.e. no death)
β	Rate of secondary infection	0.03d^-1^
ε	Rate of primary infection	0 d^-1^
*K*(*d*;*α*)	Dispersal kernel at distance *d*, scale parameter *α* (exponential or Cauchy; see (Equation 1))	Exponential
*α*	Scale of dispersal kernel	20m
*E*_*0*_	Number of hosts that become exposed at *t* = 0	2
*ω*(*t*)	Weather suitability (either (S)uitable or (U)nsuitable)	n/a
*ω*_*s*_	Relative suitability of an suitable environment	1
*ω*_*u*_	Relative suitability of an unsuitable environment (*ω*_*u*_ ≤ *ω*_*s*_)	1 (i.e. no difference)
*T*_*w*_	Time step on which suitability changes	5d
η	Probability suitable weather becomes unsuitable, p(U | S)	0.5
ρ	Probability unsuitable weather becomes suitable, p(S | U)	0.5
*T*_*s*_	Time between successive surveys	90d
*p*	Probability that symptoms are detected	0.8
*f*	Fraction of hosts that are not surveyed (i.e. fixed as undetectable at the start of each simulation)	0
*T*_*0*_	Time of first survey	0d
*L*	Cull radius around each detected host	75m
*T*_*c*_	Notice period (i.e. average time taken to initiate culling)	60d
*σ*_*c*_	Standard deviation of the notice period	0d
κ_*E*_	Epidemic impact (i.e. the total number of hosts lost to either disease or control)	n/a
${\hat{\kappa }}_{E}$	Normalised epidemic impact, i.e. ${\hat{\kappa }}_{E}=\frac{\kappa_{E}}{\max\left(\kappa_{E}\right)}$	[0,1]
τ_*E*_	Epidemic time (i.e. the time taken to eradicate the pathogen)	n/a
${\hat{\tau }}_{E}$	Normalised epidemic time, i.e. ${\hat{\tau }}_{E}=\frac{\tau_{E}}{\max\left(\tau_{E}\right)}$	[0,1]
Ψ_*E*_	Normalised epidemic cost ($\psi_{E}\text{= }\text{(1-η) }{\hat{\kappa }}_{E}\text{ + η }{\hat{\tau }}_{E}$)	n/a
η	Relative weighting of global impacts in Ψ_*E*_	[0,1]
*A*_*E*_	Area under the disease progress curve $A_{E}=\int_{t=0}^{\tau_{E}}\left(C(t)+I(t)\right)dt$	n/a
λ	Connectivity between the local and global populations	10^-5^ d^-1^
*p*_*E*_	Probability of the pathogen escaping the region of active control (*p*_*E*_=1-exp(-λ*A*_*E*_))	[0,1]
${\hat{p}}_{E}$	Normalised probability of escape, i.e. ${\hat{p}}_{E}=\frac{p_{E}}{\max\left(p_{E}\right)}$	[0,1]
ζ_*E*_	Normalised epidemic cost ($\zeta_{E}\text{= }\text{​(1-}\delta \text{) }{\hat{\kappa }}_{E}\text{ + }\delta \;{\hat{p}}_{E}$)	n/a
δ	Relative weighting of global impacts in ζ_*E*_	[0,1]

The dispersal kernel, *K*(*d*;*α*), reflects the probability that an infectious host causes infection of a susceptible host at distance *d*, and is governed by scale parameter α. To allow robustness to the form of dispersal to be explored, we consider two contrasting kernels: the thin-tailed exponential kernel, *K*(*d*;*α*) = *A* exp (–*d*/*α*), and the thick-tailed Cauchy kernel, *K*(*d*;*α*) = *A*/(1+(*d*/*α*)^2^). In each case the normalising constant *A* is set via $(1/N)\sum_{i}\sum_{j\neq i}K(d_{ij};\alpha )=1$.

### Detection and control

We assume that the host landscape is surveyed for disease at regular intervals *T*_*s*_, starting at time *t* = *T*_*0*_, and on each survey any symptomatic (i.e. class D or I) hosts are independently detected with probability *p*. Detected hosts are flagged for subsequent removal, together with any other hosts (irrespective of disease status) within a pre-determined distance *L* of each detected focus. In practice, removal actually occurs after a variable time delay, and we assume this is normally distributed with specified mean (*T*_*c*_) and standard deviation (*σ*_*c*_). This allows for logistic delay(s) in control, including notice periods to allow for legal challenges, or delays in deployment of requisite equipment and/or manpower. A truncated normal distribution is used to ensure that all delays are positive and so that removal occurs strictly after detection. Removed hosts are not replanted in our model, in keeping with the original practice for citrus canker in Florida.

### Environmental drivers

We model environmental suitability for pathogen spread via a discrete-time Markov chain with two states, (S)uitable and (U)nsuitable (Fig 1b). This controls *ω*(*t*) in (Equation 1), with *ω*(*t*) = *ω*_s_ = 1 for state S and *ω*(*t*) = *ω*_*u*_ *≤* 1 for state U. The value *ω*_*u*_ is therefore a measure of the relative unsuitability of state U. A probabilistic transition between states potentially occurs every *T*_*w*_ units of time. The probability of entering either state then depends only upon the current state, with p(U | S) = *η* and p(S | U) = *ρ*. At equilibrium, the probability of the environment being suitable (i.e. in state S) is $\pi =\frac{\rho }{\eta +\rho }$, and so the mean value of *ω*(*t*) is *π* + (1–*π*)*ω*_*u*_. The initial state is chosen randomly according to π, ensuring that the equilibrium properties of the chain control its statistics.

Markov chains offer a parsimonious approximation to environmental dynamics in a number of contexts [32], and a similar two-state formalism has previously been used to model infection rates of plant pathogens [33]. An advantage of a Markov chain model is that it simply requires information on threshold conditions, for example temperature and humidity, that favour or inhibit infection, and these are likely to be known for many plant pathogens or can at least be quantified relatively easily. Although we acknowledge more extensive information concerning these drivers is in fact already well-known for citrus canker [29], in general this obviates the need to derive costly functional forms for relationships between propagule production and environmental driving variables. By default, however, in illustrating the use of the model we restrict our attention to the case in which *ω*_*u*_ = *ω*_*s*_ = 1.

### Parameterisation

Here we use the citrus canker system as a case study to illustrate general principles underlying the effectiveness of control. Parameters can readily be adapted via our front-end interface to reflect other pathosystems (*cf*. S2 Text, which describes the application of our model to HLB, and S1–S3 Figs., which show the results). As defaults we therefore use illustrative parameters informed by the biology [29,30] and adapted from previous models of citrus canker [11,12,24,25] (Table 1) to drive our mathematical model. The host population is surveyed every 90 days [30], symptomatic hosts are detected with probability 0.8, and host removal occurs exactly 60 days after detection [29]. The default cull radius is set to be 75m; this default radius was chosen to emphasise the range of outcomes that is possible for a single control strategy, even when all parameters remain fixed. Epidemics are seeded with two exposed hosts at *t* = 0 (a different pair of hosts for each realisation). The average latent period is 10d [29] and symptoms take an average of 110d to emerge following infection [11]. The dispersal kernel is exponential, with mean dispersal distance of 40m (i.e. *α* = 20m), and we take the rate of secondary infection to be β = 0.03d^-1^ (*cf*. the values of *α* = 37m and β = 0.036d^-1^ as used in the analyses of Cook *et al*. and Parnell *et al*. [11,12,24], after accounting for our normalisation of the dispersal kernel). We selected default dispersal scale and infection rate parameters that lead to slightly slower and more spatially-restricted spread in comparison with those in previous analyses. This allows us to present extensive sensitivity analyses to parameters that would be expected to make control more difficult (e.g. long cryptic periods, lengthy delays between detection and tree removal), without optimal controls degenerating to immediate removal of the entire population at the time of the first control for our rather small population of interest. However, we demonstrate the robustness of our results to this slight alteration of the parameters in S3 Text and S4–S6 Figs., in which we repeat a selection of the analyses using exactly the parameters of Cook *et al*. [24] as a baseline.

### Online interface and software architecture

In explaining and discussing the practical use of this model with stakeholders, including policy makers, regulators and growers, it became apparent that providing a user-friendly version of the model for presentation was important to allow the inferences to be understood and visualised by non-specialists. We therefore developed an online interface to the model, allowing the results of either a single run or a small ensemble of runs to be explored, and also allowing for the alteration of parameters controlling disease spread and/or control. This Webidemics front-end runs in commonly-used web browsers via the freely-available Adobe Flash Player plug-in. It is an interface to a ‘back-end’ program that runs on a central web server; this is written in C, and is the component that actually performs the model simulations presented in this paper. Implementation of the back-end via Gillespie’s algorithm [34] allows the extensive replication (many millions of independent runs) that underlies the analytical results we present. It also allows the user of the front-end to obtain results from an entire ensemble of hundreds of replicate epidemics within a reasonable time. Parameters and results are passed between the front- and back-ends via a Perl CGI (“Common Gateway Interface”) wrapper program hosted on the web server.

## Results

### Optimal culling radius under uncertainty

**Fig 2:**
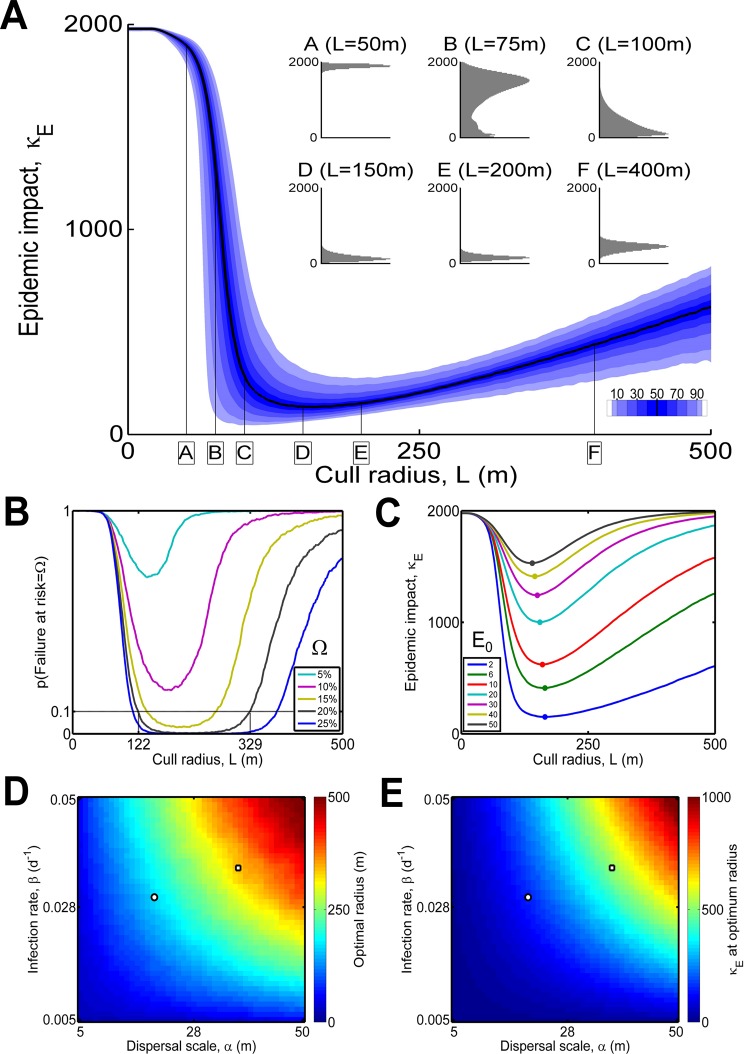
Optimal control when there is uncertainty. (a) Epidemic impact κ_*E*_ (total number of hosts lost to disease or control) as a function of the cull radius, *L*. The optimum value of *L* depends on the percentile of the distribution of κ_*E*_ that is being optimised (e.g. the optimum *L* would be 159m if the objective were to minimise median κ_*E*_, whereas it would be 194m if minimising κ_*E*_ on the 95^th^ percentile). The shape of the distribution of κ_*E*_ varies with *L* (insets A to F; distributions renormalized to the same height by scaling all distributions relative to the largest value in each). (b) Risk of failure. Given a notion of “acceptable risk” (i.e. a value of Ω, the threshold κ_*E*_ as a percentage of the total population), the probability of failing to achieve κ_*E*_ < Ω is shown. Dotted line marks radii with < 10% risk of failure for Ω = 20% (range 122m < *L* < 329m). (c) Effect of the initial level of infection, E_0_, on the response of median κ_*E*_ to *L* (dots show minimum median κ_*E*_ for each E_0_). (d) and (e) Effect of the scale of dispersal (*α*) and rate of infection (*β*) on the optimal *L* (shown in d) and median κ_*E*_ at optimal *L* to optimise median κ_*E*_ (shown in e). The white dots on (d) and (e) indicate the default values of *α* and *β*; the white squares show the values of *α* and *β* fitted by Cook *et al*. [24] (and used in the studies of Parnell *et al*. [11,12]) *cf*. S3 Text.

To examine how control performance depends on the cull radius, we performed 10000 replicate simulations for epidemics spreading according to the default parameters (*cf*. Table 1), at control radii ranging from *L* = 0m to *L* = 500m (Fig 2a). The epidemic impact, κ_*E*_ (the total number of hosts lost to disease or control by the time of eradication) is highly sensitive to the cull radius, *L*. At small *L*, the region of cryptic infection surrounding detected trees is underestimated and the disease spreads widely; at large *L*, many healthy trees are unnecessarily removed. At intermediate cull radii, performance improves markedly, and an optimum radius can be uniquely determined if the objective is phrased in terms of minimising an average of the epidemic impact, κ_*E*_. For example, median κ_*E*_ is minimised at cull radius *L* = 159m, with a median of 132 hosts removed from the total of 2000. The radius *L* = 159m is therefore optimal in the sense of previous modelling studies [11,12,24], and the “intrinsic scale of the epidemic” *sensu* Gilligan [35] has therefore been identified at this radius.

However, focusing solely on average performance ignores elements of the response of epidemic impact (κ_*E*_) to the cull radius (*L*) that may be of practical significance. The distributions shown in the inset to Fig 2 for a selection of radii (*L* = 50m, 75m, 100m, 150m, 200m and 400m) allow the following pair of provisos to this naïve optimum to be identified.

- The distribution of epidemic impact can be very sensitive to small changes in cull radius, particularly if the radius is decreased from the optimal value (compare distributions C and B with distributions D and E).
- If the objective is to minimise some other metric, e.g. the 95% percentile of the epidemic impact, κ_*E*_, thereby taking more explicit account of uncertainty, the optimum radius changes. For example, the optimum radius becomes 194m when optimising over the 95^th^ percentile of κ_*E*_, and for this radius there are 149 removals on the 50^th^ percentile and 271 removals on the 95^th^ percentile (*cf*. 132 removals on the 50^th^ percentile and 309 on the 95^th^ percentile using the cull radius 159m obtained by optimising over the median value of κ_*E*_).

In practice optimisation of any control strategy would be driven by parameters estimated for pathogen spread and epidemiology, and these would be subject to error and/or uncertainty. This tends to make the sensitivity of epidemic impact (κ_*E*_) to changes in cull radius identified in i) (above) unworkable, since an optimum strategy (distribution D) that skirts so close to failure (distribution C or more dramatically B) would almost certainly be difficult to recommend in practice. A pragmatic choice would therefore be to focus on a higher percentile of the distribution of κ_*E*_ when prescribing the control, with the particular percentile selected corresponding to the risk-aversion of the decision-maker. This approach can be formalised, by explicitly considering a risk of failure that is deemed to be acceptable (Fig 2b). In particular, given a notion of an acceptable level of risk (e.g. at most a 10% chance of κ_*E*_ corresponding to the loss of more than Ω = 20% of hosts) a range of acceptable cull radii can readily be determined. A workable strategy would then be to select a cull radius near the centre of this range. Such a combination of criteria would lead to a prescribed cull radius of around 225m here (for the default parameters, p(Failure at risk = 20%) < 0.1 for 122m < *L* < 329m).

### Effects of epidemiological parameters and logistic factors on the efficiency of intervention strategies

For simplicity, we revert to using the median epidemic impact to summarise the efficacy of intervention, although we note the criterion could be readily adjusted as described above to suit different degrees of risk aversion. The optimal cull radius *L* is surprisingly unresponsive to the initial level of infection, E_0_ (Fig 2c); the “correct” radius is virtually unaffected by the epidemic size when control starts. However, the corresponding epidemic impact increases very rapidly, and, for example, when only 2.5% of hosts are initially infected (uniformly at random), approximately 80% of hosts would eventually have to be removed before the pathogen was eradicated, even when controlling optimally. This confirms and quantifies the intuition that it is important to act quickly when confronted by a new outbreak, particularly if the initial infections are not clustered in space. The optimum cull radius and the performance when controlling optimally also depend strongly on the scale of pathogen dispersal (α) and the rate of secondary infection (β) (Fig 2d and 2e).

**Fig 3:**
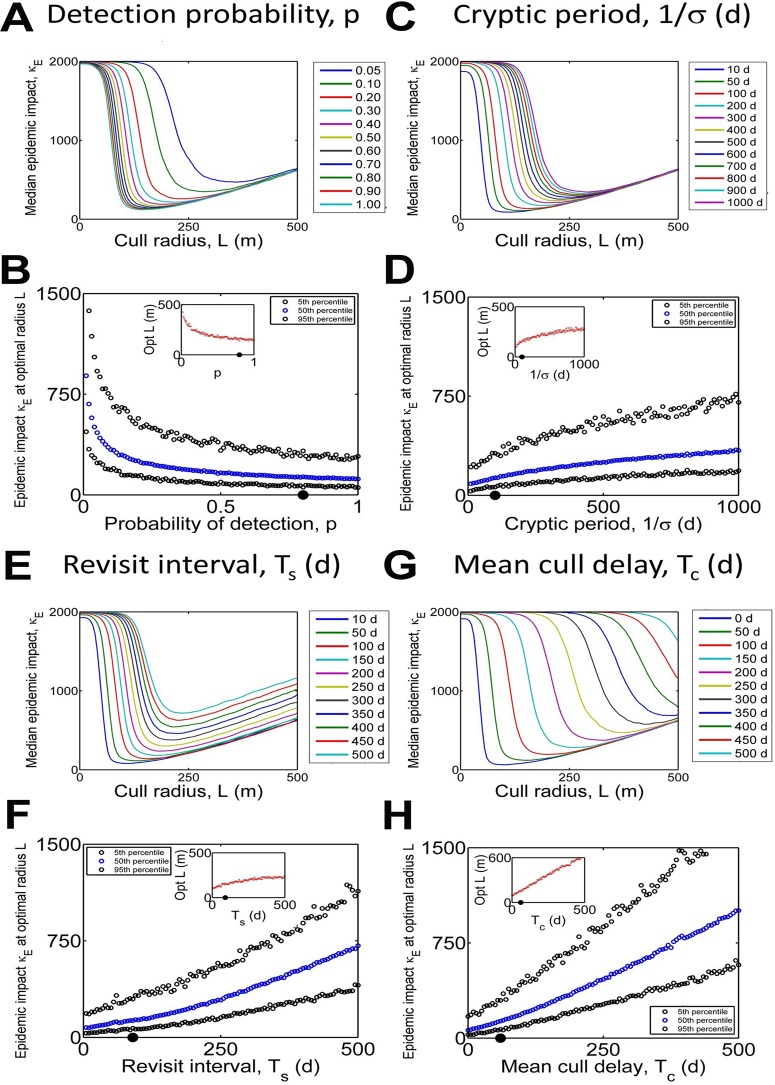
Effect of epidemiological and logistic factors on control. (a),(c),(e) and (g): Responses of median epidemic impact (κ_*E*_) to cull radius (*L*) for different values of probability of detection, *p* (a), the average cryptic period, 1/*σ* (c), the interval between successive surveys, *T*_*s*_ (e) and the notice period before culling, *T*_*c*_ (g). (b), (d), (f) and (h): How the performance of the optimum control strategy is affected by changes in *p* (b), 1/*σ* (d), *T*_*s*_ (f) and *T*_*c*_ (h). Insets show the response of the optimum cull radius *L*. Default parameter values were used for all parameters except that being scanned over: these are marked with black dots on the x-axis in (b), (d), (f) and (h).

We illustrate typical decisions that must be made by policy-makers by focusing on the effect on control performance of a selection of four parameters that may be changed during the course of an eradication scheme (Fig 3). These are the average cryptic period, (1/*σ*), which may vary depending upon environmental conditions; the probability of detecting a symptomatic host (*p*), which may vary depending on the experience of the teams of observers; and the interval between successive surveys (*T*_*s*_) and the delay period before culling actually occurs (*T*_*c*_), which are both controlled by the availability of resources. As 1/*σ*, *T*_*s*_ or *T*_*c*_ increase, or as *p* decreases, effective control becomes more challenging, and so the optimal cull radius and epidemic impact at this radius both increase. Particularly striking is that extreme changes to the probability of detection and average cryptic period are required for performance to degrade significantly. The influence of changes to either of these is mitigated by averaging over a large number of hosts: only a single host must become detectable or be detected for control to be initiated locally, and the resulting cull then affects many nearby hosts simultaneously. However, because the survey interval and the notice period both affect all hosts equally, more modest changes affect the success of control to a greater extent.

### Local versus global control

Susceptible hosts will almost always be present outside the area of first detection. However, we have focused on control performance on a small landscape; in this sense we have considered only the “local” impact of the epidemic. In practice “global” impacts (i.e. on all plants that could possibly become infected, irrespective of location) would also need attention in designing control strategies.

**Fig 4:**
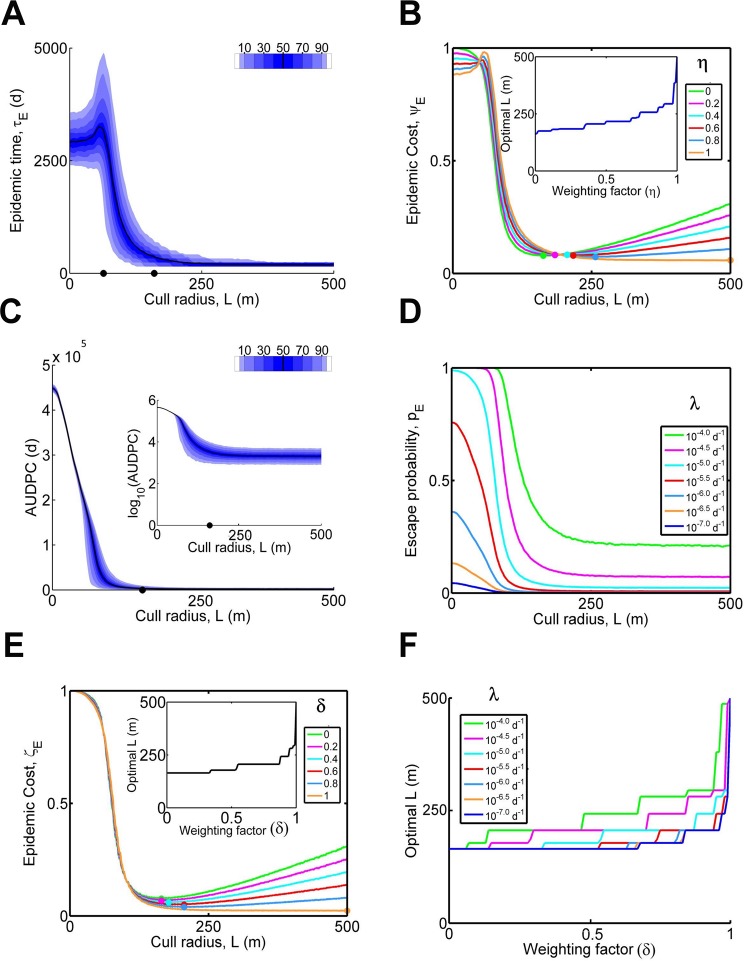
Local vs. global control. (a) Epidemic time (τ_*E*_); i.e. the time for the epidemic to be controlled, as a function of the cull radius *L*. The black dots on the x-axis mark *L* = 159m, the cull radius at which the median epidemic impact (κ_*E*_) is minimised, and *L* = 63m, the radius at which the median epidemic time (τ_*E*_) is maximised. (b) Normalised epidemic cost ($\psi_{E}\text{= ​​(1-η) }{\hat{\kappa }}_{E}\text{ + η }{\hat{\tau }}_{E}$) for a number of values of the weighting factor, η. ${\hat{\kappa }}_{E}$ and ${\hat{\tau }}_{E}$ are the epidemic impact and epidemic time normalised according to their maximum values over all cull radii considered, whereas η controls the relative weighting given to potential global impacts of the epidemic outside the area of immediate interest. The inset shows the value of *L* at which the minimum Ψ_*E*_ is obtained as a function of η. As global impacts are increasingly weighted (*η* → 1), the optimum cull radius increases, despite the increased number of local removals that would then result. (c) Area under the disease progress curve (*A*_*E*_) as a function of cull radius, *L*. The inset shows the logarithm of *A*_*E*_ as *L* is changed. (d) The probability of escape, *p*_*E*_, as function of the cull radius, *L*, for different values of the connectivity parameter λ. (e) Normalised epidemic cost ($\zeta_{E}\text{= }\text{(1-}\delta \text{) }{\hat{\kappa }}_{E}\text{ + }\delta \;{\hat{p}}_{E}$) in which the probability of escape rather than the time until eradication is included, for a number of values of the weighting factor, δ, and for fixed connectivity parameter λ = 10^-5^ d^-1^. The inset shows the value of *L* at which the optimum ζ_*E*_ is obtained for 0 ≤ δ ≤ 1. (f) Robustness to the value of λ. The inset to panel (e) is repeated for a number of values of λ; as potential global impacts are increasingly weighted (*δ* → 1), the optimal cull radius again becomes larger, for each value of λ we consider.

A possible proxy for the risk to the area outside the region of immediate interest is the time taken to eradicate the local epidemic (“epidemic time”, τ_*E*_), since this sets the duration of possible export of inoculum (Fig 4a). Surprisingly the epidemic time (τ_*E*_) initially increases as the cull radius (*L*) increases, at least for *L* below the optimum that minimises the local epidemic impact. While such controls fail to keep up with the region of cryptic infection surrounding detected hosts and so do not effectively control the epidemic, a proportion of infected plants is detected and removed on each round of surveying, and this causes the epidemic to spread more slowly (because infected hosts are being removed and there are fewer susceptible hosts to infect). Slower spread then allows the pathogen to persist for longer, since it takes more time for the infection and eventual removal of hosts. For larger control radii, however, we note the epidemic time can be very small; the pathogen is eradicated very quickly, generally within one or two rounds of detection and control.

The appropriate balance between local and global impacts is necessarily a pragmatic choice to be made by the decision maker, and it is impossible for us to be too prescriptive. We account for this need for flexibility by introducing a tuneable composite measure of global “epidemic cost”, Ψ_*E*_, intended to balance the epidemic impact and epidemic time, allowing for different weightings of local vs. global impacts. In particular, we define the normalised epidemic cost via

$$\psi_{E}\text{=}\text{(1-η)}{\hat{\kappa }}_{E}\text{+η}{\hat{\tau }}_{E},$$

where ${\hat{\kappa }}_{E}$ and ${\hat{\tau }}_{E}$ are simply the epidemic impact, κ_*E*_, and the epidemic time, τ_*E*_, normalised to a [0,1] scale (this is done by dividing by the maximum of the median values over all cull radii; for κ_*E*_ this is 1980 hosts at *L* = 0m, whereas for τ_*E*_ it is 3200d at *L* = 63m). The dimensionless weighting parameter η then controls the relative importance assigned to global impacts. In particular, taking η = 0 means only local impacts would be considered, with normalised epidemic cost $\psi_{E}\text{= }{\hat{\kappa }}_{E}$ (i.e. the normalised epidemic impact), whereas η = 1 would entirely focus on impacts outside the region of immediate interest, with $\psi_{E}\text{= }{\hat{\tau }}_{E}$ (i.e. the normalised epidemic time). As η is increased, the cull radius that minimises Ψ_*E*_ is increased (Fig 4b): the larger weighting given to global impacts means that it becomes increasingly optimal to control very aggressively to eradicate the local epidemic as quickly as possible, despite the large number of local removals that would then be required.

The epidemic time, τ_*E*_, may be of particular significance to policy makers, since the duration of a control programme will be an important determinant of public opinion. However, the time taken to eradicate the pathogen is of course not the only way of characterising the risk of pathogen spread outside the area that is actively being controlled. The area under the disease progress curve

$$A_{E}=\int_{t=0}^{\tau_{E}}\left(C(t)+I(t)\right)dt,$$

quantifies the total amount of inoculum that would be exported over the course of the entire epidemic (Fig 4c). This can be used to calculate the probability of at least one escape to the region that is not being controlled, *p*_*E*_, via

$$p_{E}=1-\text{exp}\left(-\lambda A_{E}\right),$$

where λ is a measure of the degree of connectivity between the local and non-local populations of host plants. In principle the connectivity (λ) could be determined for any particular landscape structure, although as we show here, the response of *p*_*E*_ to the cull radius (*L)* is robust to extremely wide variations in the value of λ (Fig 4d). Assuming that a single escape from the region under active control would be sufficient to initiate a global epidemic, we can then define a variant measure of epidemic cost via

$$\zeta_{E}\text{= }\text{(1-}\delta \text{) }{\hat{\kappa }}_{E}\text{ + }\delta \;{\hat{p}}_{E},$$

where δ controls the importance assigned to local vs. global impacts (i.e. δ plays the same role in the definition ζ_*E*_ of as does η in the definition of Ψ_*E*_), and where ${\hat{p}}_{E}$ is *p*_*E*_ normalised to a [0,1] scale (by dividing by the maximum value of *p*_*E*_, which occurs at *L* = 0). The response of ζ_*E*_ to the weighting parameter δ is similar to the response of Ψ_*E*_ to η (*cf*. Fig 4e, the response to different values of δ when λ = 10^-5^d^-1^), and the conclusion that the optimal radius increases with increasing the weighting of global impacts, δ, is robust to all values of the connectivity, λ, we consider, over a range of orders of magnitude (Fig 4f). Again, very extensive controls in the region under active management become optimal when the possible global impacts of disease are judged to be important.

### Thick-tailed dispersal kernels

Neri *et al*. [25] recently fitted a model of the type we use here to the dataset on the spread of citrus canker in Miami that we described in the Introduction (note this is also the dataset used by Cook *et al*. [24]). In common with Cook *et al*. [24], Neri *et al*. [25] found that an exponential dispersal kernel was best-supported by the data. However these authors found only a small difference in model goodness of fit between the exponential and Cauchy kernels. Neri *et al*. [25] suggest this partial lack of identifiability is driven by the effect of continual primary infection (i.e. infection from outside the study site, within which disease spread was mapped). At large distances from infected hosts, the small and slowly decreasing probability of infection that would be associated with Cauchy dispersal is very difficult to distinguish from the small and effectively unchanging probability that would follow an exponential kernel combined with a constant background rate of primary infection caused by fat-tailed dispersal from one or more distant sources of inoculum, or by anthropomorphic introduction of inoculum on implements, clothing or cuttings. The study sites were relatively small (<10km^2^) uncontrolled regions embedded within a large ongoing epidemic. A non-zero rate of primary infection from outside was necessary to fit the spread data in these sites, since there was significant ingress of infection from outside each site. Here, since we specifically target an isolated outbreak of emerging plant disease, far from any large source of inoculum, primary infection is not required in the analyses in the current paper. We accordingly set the rate of primary infection to zero throughout our analyses, and default to using an exponential dispersal kernel, for consistency with the fitting of Neri *et al*. [25] and Cook *et al*. [24], together with the previous analyses of Parnell *et al*. [11,12].

It is well known that exponential dispersal leads to epidemics characterised by wavelike spread, whereas thick-tailed dispersal (exemplified here by the Cauchy kernel) implies continual production of distant secondary foci with no well-defined epidemic front [36,37]. The striking difference in epidemic pattern suggests that effective control via local removal of hosts should be more difficult when there is Cauchy dispersal. For purposes of comparison, we therefore test the effect of fat-tailed dispersal on our analyses

**Fig 5:**
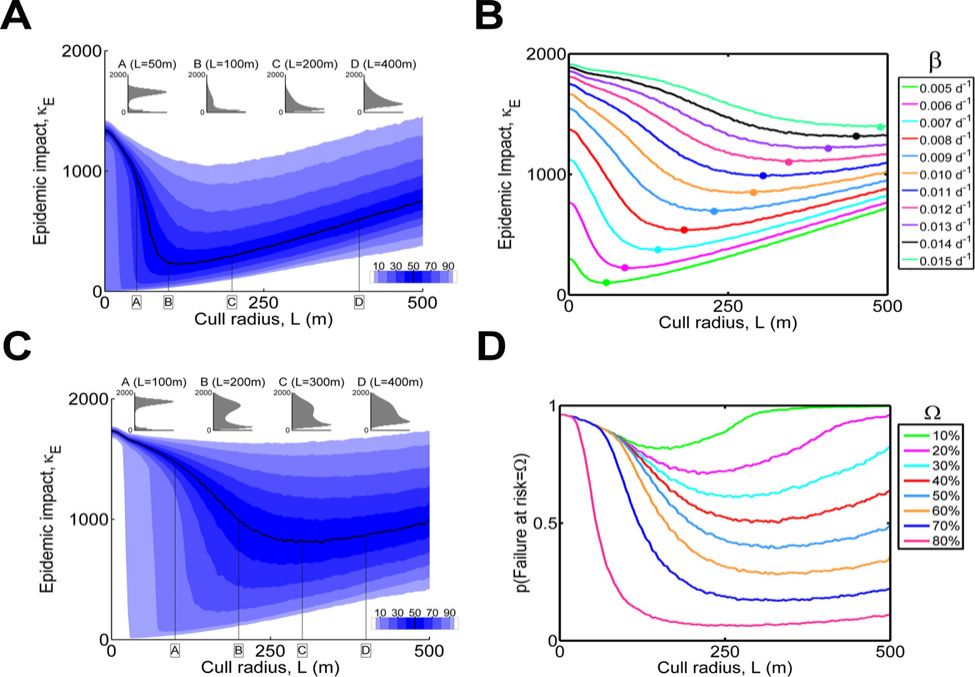
The effect of thick-tailed dispersal. (a) Response of the epidemic impact κ_*E*_ to the cull radius *L* with Cauchy dispersal and low infection rate (*β* = 0.007d^-1^). (b) Response of median κ_*E*_ to *L* for a number of values of *β*. Note how quickly the performance of control degrades with only relatively small increases to *β*. (c) Full response of κ_*E*_ to *L* when *β* = 0.01d^-1^. Note the extreme variability at even the optimal radius (e.g. *L* = 300m, inset C), and the significant probability of a large epidemic at this cull radius, even though the median κ_*E*_ is optimised. (d) Risk of failure for *β* = 0.01d^-1^: it is impossible to select a range of radii for which p(Risk < Ω) < 0.1 for Ω = 20% (*cf*. Fig 2b).

At low infection rates, effective control remains possible even with thick-tailed dispersal. For the Cauchy kernel with scale parameter *α* = 20m and infection rate *β* = 0.007d^-1^, good control can be achieved using a cull radius *L* = 100m (Fig 5a), although the long tail of the epidemic impact distribution (Inset B) reveals large epidemics are possible even when controlling optimally. However, successful control requires a small infection rate, and only moderate increases to the infection rate *β* greatly increase both the minimum median epidemic impact κ_*E*_ and the cull radius, *L*, at which this optimum κ_*E*_ is achieved (Fig 5b). What responses of the median κ_*E*_ to *L* and *β* do not reveal, however, is how quickly the chance of a large epidemic increases as the infection rate goes up. For an infection rate *β* of only 0.01d^-1^ there is a significant risk of failure of control, as indicated by the extremely variable distribution of epidemic impact for all control radii, even near the optimum cull radius *L* ~ 300m (Fig 5c). Indeed it is impossible to select a range of radii that leads to at most a 10% chance of losing less than Ω = 20% of hosts for this value of *β* (see Fig 5d and contrast with Fig 2c), reiterating the relative difficulty of control when there is fat-tailed dispersal. We also note that fat-tailed dispersal would be expected to increase the degree of connectivity between the local and non-local populations of host plants, *λ*, were we to repeat the analysis associated with Fig 4 using such a dispersal kernel. Taken together these observations suggest that successful control by culling may be difficult for plant pathogens that spread via windborne propagules which are tolerant to desiccation. These include powdery mildews and rust pathogens. Effective control of these pathogens is likely to require large local cull radius and could be expected to have relatively high risks of failure.

### Webidemics online user-interface

**Fig 6:**
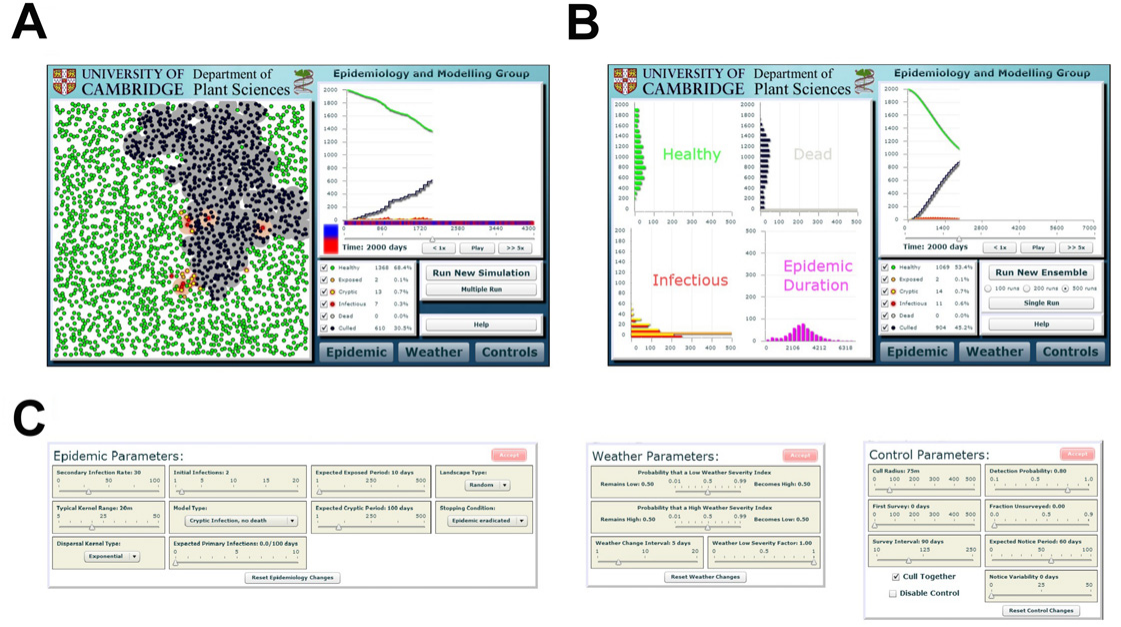
The online front-end (available at http://www.webidemics.com/). (a) A single realisation. (b) An ensemble of realisations. (c) Parameter selection. The time axis of the graph in the single realisation view colour codes environmental conditions (suitable for transmission is red, unsuitable is blue).

The Webidemics interface (http://www.webidemics.com/) shows either the results of a single realisation of the model (Fig 6a) or summarises an entire ensemble of replicate simulations performed using identical parameters (Fig 6b). When results from a single run are displayed, an animation of disease progress is shown, with hosts colour coded by epidemiological type (i.e. S, E, C or D, I, R), and with any hosts set to be inaccessible for detection denoted by a black cross. Any hosts in compartment R that were removed by disease-induced death are distinguished from those culled by control. The same colour coding is used for the graph showing the number of hosts in each class.

In the single run screen, the graph shows the time-dependence in the number of hosts in each class for the realisation being shown. However, in the ensemble of runs screen, the graph tracks the time-dependence of the average number of hosts in each compartment. The left hand panel then shows animated histograms of the numbers of hosts in each compartment over time. Clicking on any bar in a histogram switches back to the single run view, displaying a (randomly chosen) realisation from within the original ensemble that had a number of hosts within the range of the chosen histogram entry at the relevant time.

Epidemiological, climatic and/or control parameters may be set on either screen, using the three buttons at the bottom right: clicking any of these buttons reveals a pop-up panel allowing parameter values to be set (Fig 6c). When parameters are altered, the displayed results are not updated until a call is made to the back-end to actually run new simulation(s) with the new parameters (this is done by clicking the “Run New Simulation”/“Run New Ensemble” button). Changes to parameters that have not yet been followed by a call to the back-end and so are not reflected in the current results are indicated by a colour change of the button from grey to red. Further details of the user-interface are available via its help facility, which includes a full description showing how to use model in practice, designed for first time users.

### Illustrative example of the results as seen via the front-end interface

**Fig 7:**
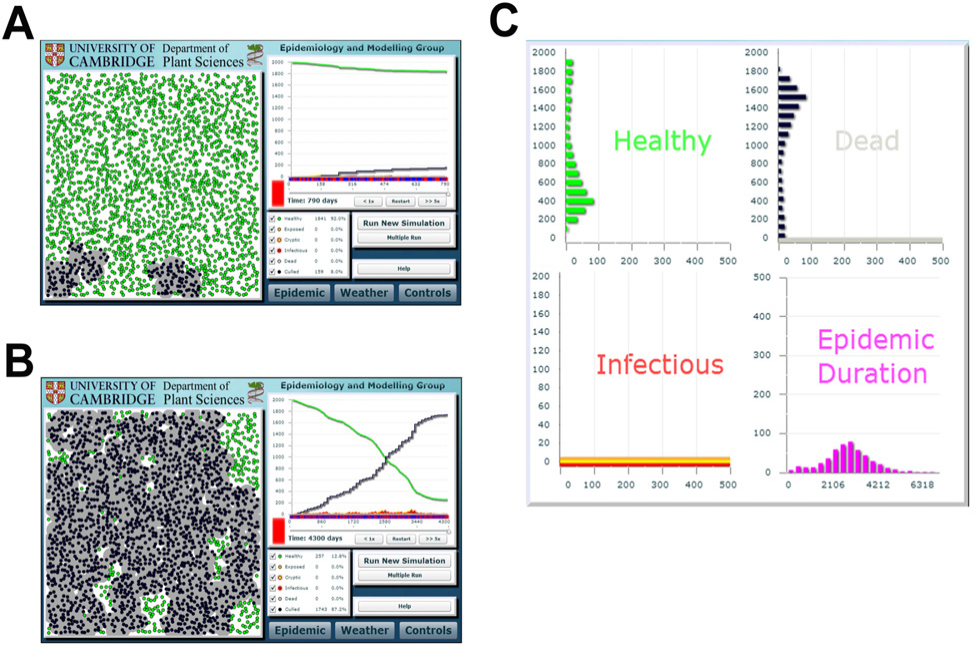
Stochasticity and the effectiveness of a single control scenario as presented via the Webidemics interface. (a) Successful control, with fewer than 10% of hosts removed before the pathogen was eradicated after 790 days. (b) A larger and more long-lasting epidemic, with ~90% removal before eradication at 4300 days. Parameters controlling pathogen epidemiology and the control intervention were the same for both runs (default values; Table 1). (c) Histograms summarising the final state in an ensemble of 500 runs (of which the individual simulation runs shown in (a) and (b) were part); note the variability in the number of healthy hosts unaffected by disease or control by the time of eradication.

The default cull radius of 75m as used in the front-end was chosen to emphasise how stochasticity can affect the effectiveness of a single control scenario when all parameters are fixed. We show here the results from this scenario, using fixed default epidemiological and control parameters (see Table 1), as seen by the user of our front-end interface. Control efficacy is extremely variable (Fig 7a and 7b). The realisation shown in Fig 7a leads to fewer than 10% of hosts (159 from 2000) removed before eradication at 790 days. However the simulation run shown in Fig 7b reveals the risk of a far greater epidemic impact, despite identical parameters controlling disease spread and control. A small proportion of asymptomatic but infectious trees escape control on each round of removal, and this leads to widespread disease. Nearly 90% of hosts (1743 out of 2000) are eventually removed before the pathogen is fully controlled at *t* = 4300d. Similar behaviour is easily observable via the interface (*cf*. Fig 7c, which shows typical histograms summarising the final state of 500 runs using the default parameterisation).

## Discussion

We present a novel stochastic analysis of the control of plant disease, focusing on how the performance of reactive control by localised culling can be optimised. We have also introduced Webidemics, an interactive online tool designed to communicate principles affecting effective control to an audience of stakeholders, including policy makers, regulators, growers and scientists. Default parameterisation of the underlying epidemiological model targets the spread of citrus canker in Florida by adapting the parameters of previous modelling studies, although these defaults can readily be altered to represent other model parameterisations or even pathosystems by the user of our front-end interface. The analysis and the user-friendly interface address and illustrate the challenges posed by cryptic infection, stochasticity and uncertainty in parameter values, and demonstrate how these factors must be accounted for in designing successful disease control strategies.

Our key result is to verify that it is indeed possible to optimise control via targeted host removal by matching the “intrinsic scale of the epidemic” [35], selecting a cull radius that minimises the epidemic impact, κ_*E*_ (i.e. the total number of hosts lost to disease or control before the pathogen is eradicated). However, given particular parameters controlling disease spread and the logistics of control, we have shown how the cull radius that would be selected depends on the percentile of the epidemic impact distribution that is to be optimised over, and therefore on the risk-aversion of the decision-maker. Since costs of disease are typically greater than costs of detection and control, under-control can be more harmful than over-control [22]. This is reflected in the sharp increase in epidemic impact, with even small decreases in the cull radius below the optimum (Fig 2), a pattern that is largely unresponsive to the values of the parameters (Fig 3). The pattern also holds for other baseline sets of parameters (S3 Text) and also when applying the model to other pathosystems (S2 Text). We have also confirmed the intuition that control should start quickly to be successful; if even a small proportion of hosts is infected when intervention commences, a large epidemic impact appears unavoidable, particularly if the initial infections are not spatially-clustered (Fig 2c).

We investigated how the nature of the optimum control strategy is conditioned on a selection of parameters controlling the host-pathogen interaction and the logistics of control. Our results show how success of control depends strongly on the rate and spatial scale of pathogen spread (Fig 2d and 2e). We also showed how factors that act differently at the level of the individual host can have less severe effects as they are altered than those that affect all individuals equally, as a consequence of the former being averaged over the entire population (Fig 3). While control by local culling can remain viable when there is thick-tailed dispersal, control is only then successful for low infection rates (Fig 5). Even moderate rates of spread due to higher infection rates (β) lead to too many new disease foci when there is fat-tailed dispersal, and, in turn, this means that control is often unsuccessful, with significant risk of large epidemic impacts. This high risk of failure remains hidden if only the median epidemic impact is considered (Fig 5c), re-emphasising the need to examine the full distribution of outcomes when assessing the efficacy of control.

Our purpose here was to illustrate and test some key principles underlying the success of control. For this we selected a biologically-plausible set of parameters for citrus canker that allowed extensive sensitivity analyses to factors that are likely to affect the success of control. Our initial analyses focused on the optimal cull radius to eradicate disease in local outbreaks following a small number of primary infections in a spatially restricted (2km x 2km) urban host landscape. Our analyses show that the magnitude of the optimal cullradius depends upon the degree of risk aversion to failure of control, calculated from percentiles for the probability of a given epidemic impact that accounts for the cost of disease and the cost of control. Values for our default parameterisation for citrus canker varied from 104m for the 5^th^ percentile of local epidemic impact to 194m for the 95^th^ percentile, with an optimal radius of 159m when optimising over the median impact (Fig 2). However, while the responses of epidemic impact to changes in the cull radius, and of the optimum cull radius to different levels of risk aversion, are both robust, the basic estimate of the optimal cull radius can vary widely (from ~100m to ~500m), depending on changes to epidemiological and logistical parameters (Fig 3) and the underlying parameterisation selected for the model (S3 Text).

All of these figures relate to local control and are typically smaller than the cull radius of 571.9m used by USDA for statewide control of the citrus canker epidemic in Florida from 2002 [29]. One driver of this difference is that our default host landscape uses planting densities typical of residential citrus, whereas extended regions of commercial citrus would lead to faster spread due to higher planting densities. Moreover uncooperative landowners meant that in practice certain trees were inaccessible for pathogen survey, and legal challenges sometimes led to extremely long delays before other trees could be cut down. While both of these may be investigated via the front-end, our default parameters arguably downplay these effects (e.g. a fixed notice period of 60 days, when in practice legal challenges could lead to delays of many months or even years). More significantly, however, these initial estimates do not take account of the risk of infection spreading from the prescribed region of interest to surrounding regions. As we have shown here, to advance from optimisation at local to statewide scales in fact requires proper consideration of the balance between local and global impact of the epidemic (Fig 4).

Work targeting animal epidemics suggests optimal control strategies depend strongly on how local and global impacts are balanced [21,22]. We examined this by introducing two variants of the “epidemic cost”, that account for possible infections outside the area of interest via the proxies of local time to eradication or the probability of pathogen escape, but that are flexible enough to allow for different weightings of local vs. global priorities. Strategies giving a high weighting to global performance required extensive controls at the local level (Fig 4b, 4e and 4f). This is because the potential for spread of disease to create a new focus of infection elsewhere is judged to be so harmful that it becomes optimal to use a rather draconian policy in the region under active control, even at the cost of many local removals.

While we motivate our analyses using citrus canker, our work can readily be placed in a broader context. The recent emergence of citrus greening or huanglongbing (HLB, caused by *Candidatus Liberibacter* spp. bacteria), potentially an even more devastating disease [38], puts control of citrus pathogens firmly back on the scientific and political agenda in the United States. HLB is vectored by psyllids, and although it would be reasonable to assume dispersal of infective vectors declines monotonically with distance from infected plants, it might be expected that changes in psyllid populations over time would add extra complexity to disease dynamics. However, recent work has shown how our underlying model focusing only on disease status and representing disease spread via a time-independent and spatially-isotropic dispersal kernel can be applied to this pathosystem with no change to the fundamental model structure [26]. We recreated a selection of our results for the control of HLB in a citrus grove in S2 Text. We emphasise that using this type of model for HLB means that the activity and population dynamics of the psyllid population are not tracked explicitly, but instead that these factors are included in the dispersal and infection rate parameters of the model. The results concerning principles for control were qualitatively unchanged, although of course the exact detail of the optimum radius and epidemic impact were different reflecting a different pathogen and host topology.

Similar models are increasingly used for other plant pathogens at both small [11–13,18,24–26,39–42] and large spatial scales [10,16,36,43–47]. An emerging epidemic to which models are already being applied is sudden oak death (caused by *Phytophthora ramorum*) in the United Kingdom [48]: predictions from a larger-scale stochastic compartmental model are already informing the extent of felling of commercial larch [49]. The United Kingdom government’s response to Chalara ash dieback is also based on predictions from this type of model [50]. Models with static hosts have also been applied to pathogens of agricultural animals, most notably for epidemics of foot and mouth disease.

Our particular focus has been a simple control strategy, in which all hosts within a certain distance of detected hosts are removed, and where this distance is fixed in advance. While this corresponds to the approach most often taken in practice, and has definite advantages in terms of ease of implementation and transparency to those affected by control, recent modelling work has examined more elaborate strategies. In particular te Beest *et al*. [51] consider a complex and time-varying control strategy for an animal disease epidemic spreading through a set of farms that takes account of heterogeneity in the potential risk according to farms’ position and the current state of the epidemic. Our results are also conditioned on the metric used to define the epidemic impact. More complex notions of cost are possible; an obvious extension, for example, would be to include the cost of detection [14,15,18]. Although our model allows for fluctuations in environmental conditions and we allow the user to set parameters causing the pathogen to be affected by the environment via the front-end, we have not focused on these effects in this paper. We have also not accounted for the additional and significant difficulty in control of novel invasive pathogens for which the parameters controlling spread are themselves ill-characterised. Nor have we considered the effects of any spatial patterning of the host population in terms of, for example, systematic differences in host quality or differential resistance, although we note these spatial patterns would need to be well-characterised in order to be used in the model. We suggest that exploration of these issues, together with assessing and optimising the performance of control scenarios on spatially-extended host landscapes and particularly when there is thick-tailed dispersal, are important challenges. Further work is also underway to augment our theoretical work with interactive user-friendly front-end interfaces, after our extremely positive experiences in using the Webidemics interface to explain and present the ideas underlying our results to an audience of non-specialists.

## Supporting Information

S1 Text Information on the attempted eradication of citrus canker from Florida between 1996 and 2006. (DOCX)

S2 Text Application of the model to a vectored pathogen: control of an isolated outbreak of huanglongbing disease in commercial citrus. (DOCX)

S3 Text Results for citrus canker using the dispersal scale and infection rate parameters as presented by Cook *et al*. [24]. (DOCX)

S1 Fig Using the online front-end interface for HLB. Screenshots showing which parameters need to be changed in the front-end to recreate the analysis for the spread of HLB in a citrus grove (using the parameterisation presented by Parry *et al*. [26]) as described in S2 Text (a) epidemic parameters; (b) control parameters. The parameters that must be changed are highlighted in pink.
(TIF)

S2 Fig Optimal control and the effect of uncertainty for HLB. (a) Epidemic impact κ_*E*_ (total number of hosts lost to disease or control) as a function of the cull radius, *L*. This replicates Fig 2a in the main text for the HLB system. (b) Risk of failure. Given a notion of “acceptable risk” (i.e. a value of Ω, the threshold κ_*E*_ as a percentage of the total population), the probability of failing to achieve κ_*E*_ < Ω is shown. Dotted line marks radii with < 10% risk of failure for Ω = 40% (range 32m < *L* < 90m). This is equivalent to Fig 2b in the main text for the HLB system rather than citrus canker.
(TIF)

S3 Fig Effect of epidemiological and logistic factors on control for HLB. (a),(c),(e) and (g): Responses of median epidemic impact (κ_*E*_) to cull radius (*L*) for different values of probability of detection, *p* (a), the average cryptic period, 1/σ (c), the interval between successive surveys, *T*_*s*_ (e) and the time at which detection starts, *T*_*0*_ (g). (b), (d), (f) and (h): How the performance of the optimum control strategy is affected by changes in *p* (b), 1/σ (d), *T*_*s*_ (f) and *T*_*0*_ (h). Insets show the response of the optimum cull radius *L*. Default HLB parameter values (*cf*. S1 Fig) were used for all parameters except that being scanned over: these are marked with black dots on the x-axis in (b), (d), (f) and (h). This is equivalent to Fig 3 in the main text for the HLB system rather than citrus canker.
(TIF)

S4 Fig Using the online front-end interface to replicate the analysis using the parameterisation of Cook *et al*. [24]. Screenshots showing which parameters need to be changed in the front-end to recreate the analysis using the parameterisation originally developed by Cook *et al*. [24] and used in the subsequent analyses by Parnell *et al*. [11,12]. The pair of parameters that must be changed are highlighted in pink.
(TIF)

S5 Fig Optimal control and the effect of uncertainty using the parameterisation of Cook *et al*. [24]. (a) Epidemic impact κ_*E*_ (total number of hosts lost to disease or control) as a function of the cull radius, *L*. This replicates Fig 2a in the main text using the parameterisation originally developed by Cook *et al*. [24] and used in the subsequent analyses by Parnell *et al*. [11,12]. (b) Risk of failure. Given a notion of “acceptable risk” (i.e. a value of Ω, the threshold κ_*E*_ as a percentage of the total population), the probability of failing to achieve κ_*E*_ < Ω is shown. Dotted line marks radii with < 10% risk of failure for Ω = 50% (range 287m < *L* < 549m). This is equivalent to Fig 2b in the main text but using the parameterisation originally developed by Cook *et al*. [24] and used in the subsequent analyses by Parnell *et al*. [11,12].
(TIF)

S6 Fig Effect of epidemiological and logistic factors on control using the parameterisation of Cook *et al*. [24]. (a),(c),(e) and (g): Responses of median epidemic impact (κ_*E*_) to cull radius (*L*) for different values of probability of detection, *p* (a), the average cryptic period, 1/σ (c), the interval between successive surveys, *T*_*s*_ (e) and the notice period before culling, *T*_*c*_ (g). (b), (d), (f) and (h): How the performance of the optimum control strategy is affected by changes in *p* (b), (d), *T*_*s*_ (f) and *T*_*c*_ (h). Insets show the response of the optimum cull radius *L*. The parameter values fitted by Cook et al. [24] (*cf*. S4 Fig) were used for all parameters except that being scanned over: these are marked with black dots on the x-axis in (b), (d), (f) and (h). This is equivalent to Fig 3 in the main text but using the parameterisation originally developed by Cook *et al*. [24] and used in the subsequent analyses by Parnell *et al*. [11,12].
(TIF)
