# Supplementary material for: Optimising and Communicating Options for the Control of Invasive Plant Disease When There Is Epidemiological Uncertainty
Source: PLoS Comput Biol. 2015 Apr 13;11(4):e1004211. doi: 10.1371/journal.pcbi.1004211 (PMC4395213; doi:10.1371/journal.pcbi.1004211)
Supplement: S1 Text — (DOCX) [file pcbi.1004211.s001.docx]

**S1 Text**

**Attempted eradication of citrus canker from Florida between 1996 and 2006**

Citrus canker, caused by the bacterium *Xanthomonas citri* pv. *citri*, is a disease of citrus characterised by erumpent lesions on fruit, foliage and young stems, to which most commercial citrus varieties are moderately to highly susceptible. The most recent introduction to Florida is estimated to have occurred in 1993, with the first detection of the pathogen near Miami airport in 1995 [1]. The US government spent an estimated $1 billion on survey, control and compensation costs during a nine year campaign that attempted to eradicate citrus canker [2]. This programme resulted in the removal of at least ten million citrus trees across the state of Florida [3], from both commercial citrus groves and residential settings.

Attempted eradication was based on regular surveys, aimed at detecting symptomatic trees, followed by removal of all “exposed” trees, which in this context corresponds to trees judged to have a high probability of being infected [4]. Assessment of trees as exposed was controlled by a simple proximity rule: all host trees within a particular radius of a known infected tree were assumed to be exposed. The cull radius was initially set to be 38.1m (125 ft) [5], but was increased to 579.1m (1900 ft.) in 2002 [2], after it became obvious that the initial radius was not successfully retarding spread. A potential driver that is widely acknowledged to have become increasingly important as the epidemic progressed, and that was used to motivate the increased cull radius, is the Asian citrus leafminer (*Phyllocnistis citrella*). This facilitates bacterial infection by wounding host organs and thereby greatly increases potential spread [2].

The eradication programme was highly controversial. Certain groups found it extremely difficult to understand why apparently healthy trees should have to be removed. In particular, a group of residents of south Florida obtained an injunction to stop removal of non-infected citrus in May 2002, arguing that commercial citrus was being protected at the expense of residential trees. Although the ruling was overturned in January 2003, this moratorium halted the programme for approximately eight months, which in turn facilitated increased pathogen dispersion [6]. Hurricanes and tropical storms in 2004, and (particularly) Hurricane Wilma in October 2005, led to the further dispersal of inoculum over an extremely wide area [4]. The consensus by the end of 2005 was that the disease had spread too far and to too many locations, and that as the pathogen was endemic, eradication had become unfeasible. The programme was therefore halted in May 2006. However, litigation attempting to force the Florida Department of Agriculture to pay compensation to homeowners continues to this day [6].

**References**

1. Graham JH, Gottwald TR, Cubero J, Achor DS (2004) Xanthomonas axonopodis pv. citri: factors affecting successful eradication of citrus canker. Mol Plant Pathol 5: 1–15.

2. Gottwald TR, Graham JH, Schubert TS (2002) Citrus canker: the pathogen and its impact. Plant Heal Prog.

3. Irey M, Gottwald TR, Graham JH, Riley TD, Carlton G (2006) Post-hurricane analysis of citrus canker spread and progress towards the development of predictive model to estimate disease spread due to catastrophic weather events. Plant Heal Prog.

4. Gottwald TR, Irey M (2007) Post-hurricane analysis of citrus canker II: predictive model estimation of disease spread and area potentially impacted by various eradication protocols following catastrophic weather events. Plant Heal Prog.

5. Schubert TS, Rizvi SA, Sun X, Gottwald TR, Graham JH, et al. (2001) Meeting the challenge of eradicating citrus canker in Florida – Again. Plant Dis 85: 340–356.

6. Gottwald TR (2007) Citrus canker and citrus huanglongbing. Two exotic bacterial diseases threatening the citrus industries of the Western Hemisphere. Outlooks Pest Manag 18: 274–279.
