# Supplementary material for: Optimising and Communicating Options for the Control of Invasive Plant Disease When There Is Epidemiological Uncertainty
Source: PLoS Comput Biol. 2015 Apr 13;11(4):e1004211. doi: 10.1371/journal.pcbi.1004211 (PMC4395213; doi:10.1371/journal.pcbi.1004211)
Supplement: S2 Text — (DOCX) [file pcbi.1004211.s002.docx]

**S2 Text**

**Control of an isolated outbreak of huanglongbing disease in commercial citrus**

Recent work by Parry *et al.* [1] fitted a series of individual-based compartmental models of the type we use here to a dataset tracking spread of huanglongbing (HLB) within a large commercial citrus plantation. This allows us to test the robustness of our main results to using exactly the model presented in the main text for a different pathosystem on a different host topology.

For trees that are 10 - 20 years old, Parry *et al.* [1] state the latent period is approximately 0.8 years, and the cryptic period approximately 0.25 years. We therefore set $1/\gamma$ = 292d and $1/\sigma$ = 90d in our model. They found the scale of dispersal and rate of infection differed across the plantation between citrus blocks on a block-by-block basis, largely driven by the age of the trees within each block (their Figs. 3 and 4). Taking a representative block of 10-20 year old trees leads to a representative dispersal scale parameter of $\alpha$ = 10m (note their scale parameter is the inverse of ours), and, after accounting for their different normalisation of the dispersal kernel, a rate of infection $\beta$ = 0.085d^-1^.

As in the main text, we consider an isolated outbreak far from any ongoing epidemic, for which there is no primary infection (i.e. $\varepsilon$ = 0 in our model). By default, and to reflect the more intensive control of a managed citrus grove, we also assume that i) the citrus grove is surveyed every month (i.e. *T_s_* = 30d), ii) there is no delay between detection and removal of symptomatic trees (i.e. *T_c_* = 0d). The lack of delay before control can be applied is more realistic for commercial citrus: in the main paper we took *T_c_* = 60d for the citrus canker example, but this was to allow for legal challenges by homeowners. All other parameters were set to the values in the main text (*cf.* Table 1). Note that these analyses can therefore be recreated by the user of our front-end interface (*cf.* S1 Fig., which shows exactly which parameters must be changed via our interface). We note that the control we consider, i.e. removal of trees surrounding known infection, is not routinely practiced for HLB control in commercial citrus groves, but we consider it here to illustrate the flexibility of our models, methods and analyses for a different system.

We recreated a selection of our analyses for the HLB system. It was still possible to choose an optimal cull radius which minimises the epidemic impact (S2a Fig.; *cf.* Fig. 2a in the main text), although the exact detail of the optimum radius depends on the level of risk aversion (S2b Fig.; *cf.* Fig. 2b in the main text). The responses of the optimum radius and the epidemic size at optimum radius to changes in the parameters were similar to before (S3 Fig.; *cf.* Fig. 3 in the main text). The qualitative results presented in the main text are therefore robust to using our model and methods of analyses on a different pathosystem, here for a disease that is vectored by insects.

**References**

1. Parry MF, Gibson GJ, Parnell S, Gottwald TR, Irey MS, Gast T, et al. Bayesian inference for an emerging arboreal epidemic in the presence of control. Proc Natl Acad Sci U S A. 2014;111: 6258–6262.
