# Supplementary material for: Optimising and Communicating Options for the Control of Invasive Plant Disease When There Is Epidemiological Uncertainty
Source: PLoS Comput Biol. 2015 Apr 13;11(4):e1004211. doi: 10.1371/journal.pcbi.1004211 (PMC4395213; doi:10.1371/journal.pcbi.1004211)
Supplement: S3 Text — (DOCX) [file pcbi.1004211.s003.docx]

**S3 Text**

**Results for citrus canker using the dispersal scale and infection rate parameters as presented by Cook *et al.*** [1]

Cook *et al.* [1] fitted different values of the infection rate and dispersal scale parameters when compared with the analyses we present in the main text. The Cook *et al.* [1] parameters were subsequently used in the studies of Parnell *et al.* [2,3]. After accounting for our different normalisation of the dispersal kernel and the fact their dispersal scale parameter is the inverse of ours, these three studies all used parameters corresponding to $\alpha$ = 37m and $\beta$ = 0.036d^-1^ in our model. We recreated a selection of our analyses using these default values of the infection rate and dispersal scale parameters, with all other parameters set to the values in the main text (*cf.* Table 1 in the main text). Note that these analyses can be recreated by the user of our front-end interface (*cf.* S4 Fig., which shows exactly which parameters must be changed).

Using the Cook *et al.* [1] parameter values led to a two-fold change in our baseline estimate of the optimal cull radius in comparison with the parameterisation adopted in the analysis in the main paper (*cf.* S5 and S6 Figs.), largely due to the increased rate of pathogen spread (*cf.* Figs. 2d and 2e in the main text, where the optima of the two models are compared). However, there were similar responses of the optimal radius and the epidemic impact in response to changes to the epidemiological and logistical parameters.

**References**

1. Cook AR, Gibson GJ, Gottwald TR, Gilligan CA (2008) Constructing the effect of alternative intervention strategies on historic epidemics. J R Soc Interface 5: 1203–1213.

2. Parnell S, Gottwald TR, van den Bosch F, Gilligan CA (2009) Optimal strategies for the eradication of asiatic citrus canker in heterogeneous host landscapes. Phytopathology 99: 1370–1376.

3. Parnell S, Gottwald TR, Gilligan CA, Cunniffe NJ, van den Bosch F (2010) The effect of landscape pattern on the optimal eradication zone of an invading epidemic. Phytopathology 100: 638–644.
