# Supplementary figures and images for: Optimising and Communicating Options for the Control of Invasive Plant Disease When There Is Epidemiological Uncertainty
Source: PLoS Comput Biol. 2015 Apr 13;11(4):e1004211. doi: 10.1371/journal.pcbi.1004211 (PMC4395213; doi:10.1371/journal.pcbi.1004211)

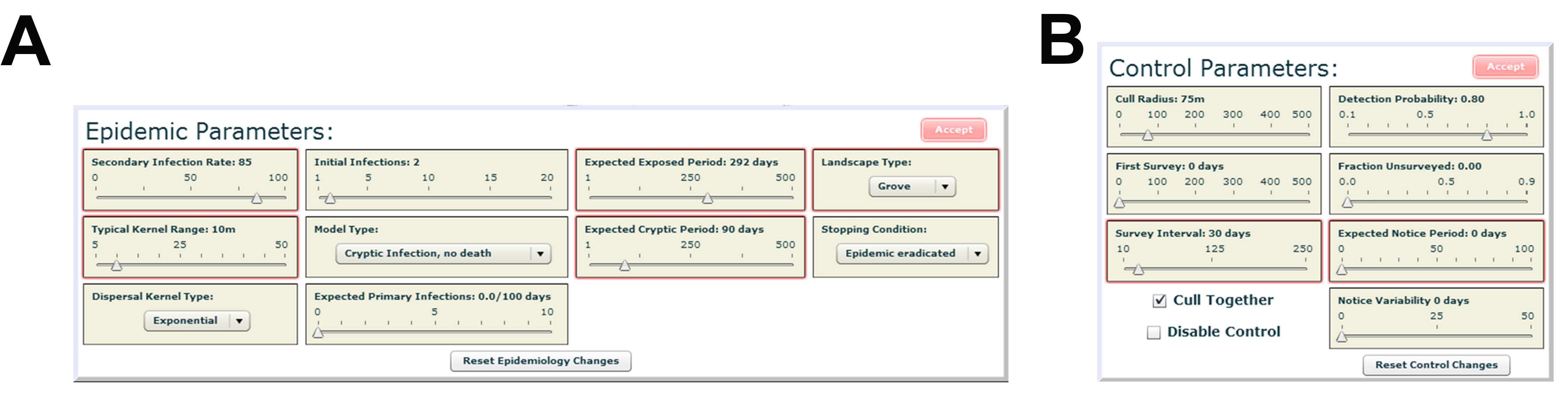

Supplement: S1 Fig — Screenshots showing which parameters need to be changed in the front-end to recreate the analysis for the spread of HLB in a citrus grove (using the parameterisation presented by Parry et al. [26]) as described in S2 Text (a) epidemic parameters; (b) control parameters. The parameters that must be changed are highlighted in pink. (TIF) [file pcbi.1004211.s004.tif]

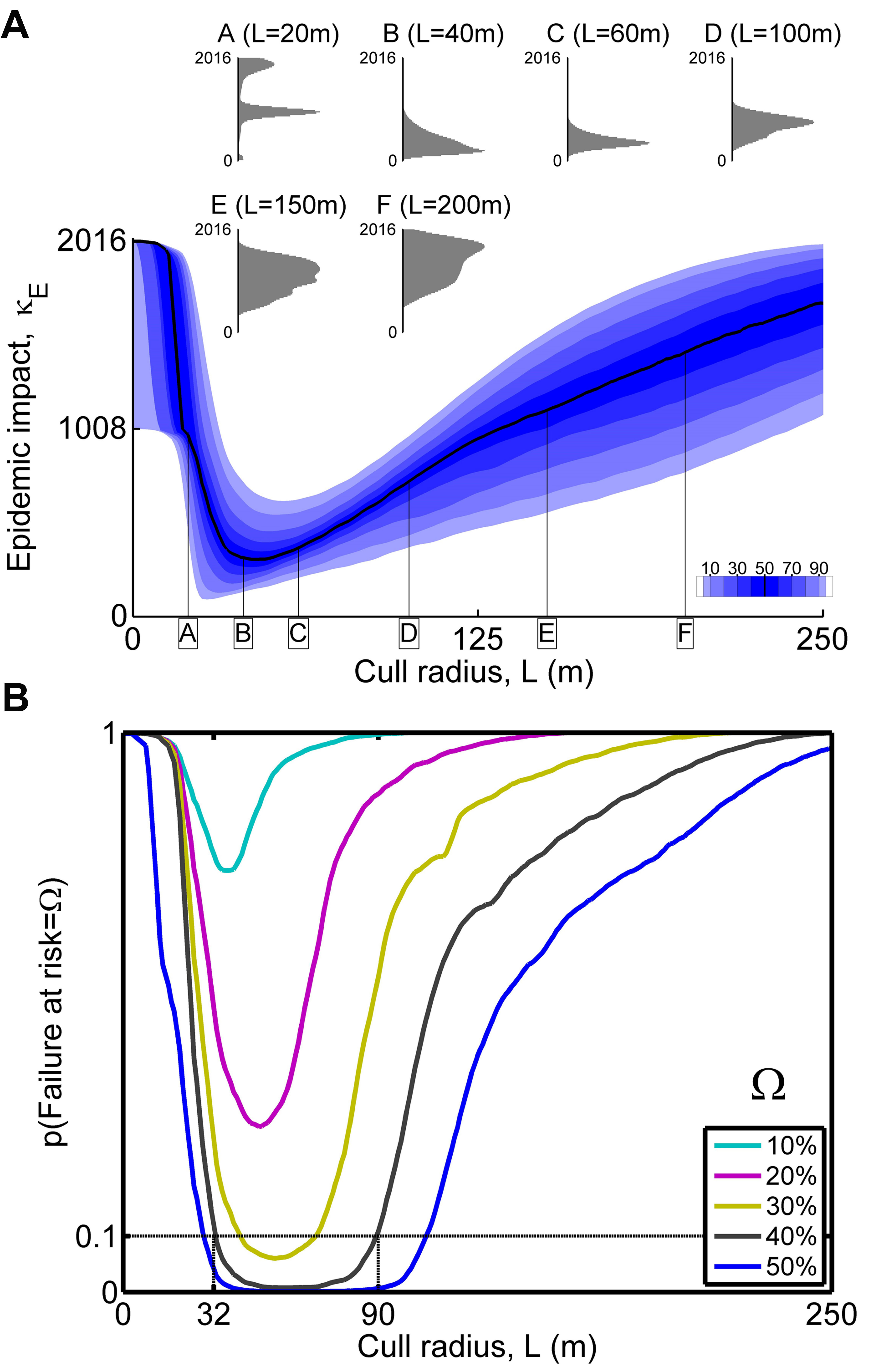

Supplement: S2 Fig — (a) Epidemic impact κE (total number of hosts lost to disease or control) as a function of the cull radius, L. This replicates Fig 2a in the main text for the HLB system. (b) Risk of failure. Given a notion of “acceptable risk” (i.e. a value of Ω, the threshold κE as a percentage of the total population), the probability of failing to achieve κE < Ω is shown. Dotted line marks radii with < 10% risk of failure for Ω = 40% (range 32m < L < 90m). This is equivalent to Fig 2b in the main text for the HLB system rather than citrus canker. (TIF) [file pcbi.1004211.s005.tif]

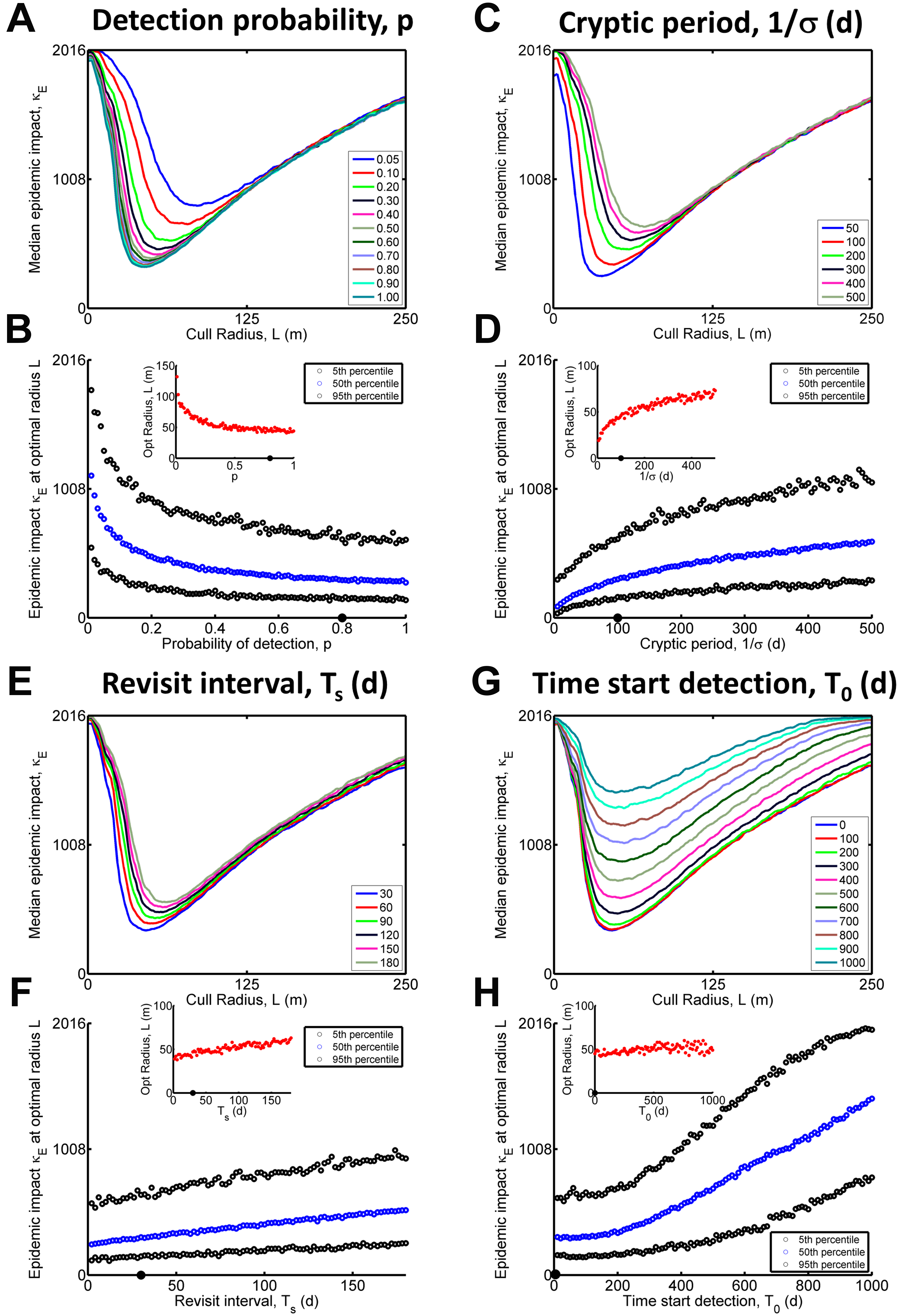

Supplement: S3 Fig — (a),(c),(e) and (g): Responses of median epidemic impact (κE) to cull radius (L) for different values of probability of detection, p (a), the average cryptic period, 1/σ (c), the interval between successive surveys, T s (e) and the time at which detection starts, T 0 (g). (b), (d), (f) and (h): How the performance of the optimum control strategy is affected by changes in p (b), 1/σ (d), T s (f) and T 0 (h). Insets show the response of the optimum cull radius L. Default HLB parameter values (cf. S1 Fig) were used for all parameters except that being scanned over: these are marked with black dots on the x-axis in (b), (d), (f) and (h). This is equivalent to Fig 3 in the main text for the HLB system rather than citrus canker. (TIF) [file pcbi.1004211.s006.tif]

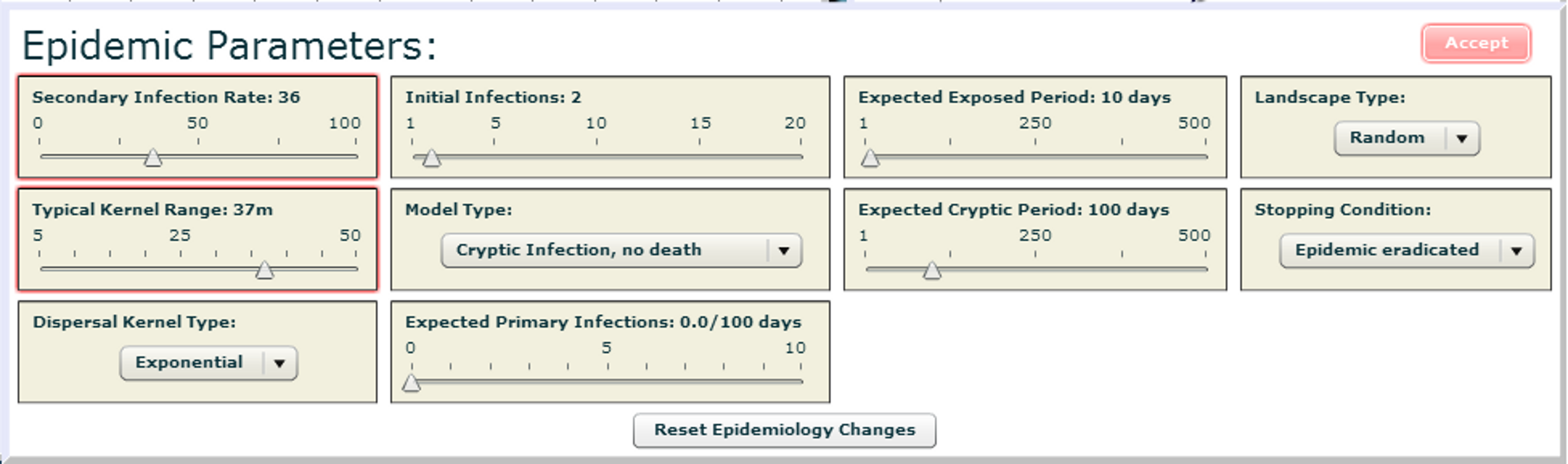

Supplement: S4 Fig — Screenshots showing which parameters need to be changed in the front-end to recreate the analysis using the parameterisation originally developed by Cook et al. [24] and used in the subsequent analyses by Parnell et al. [11,12]. The pair of parameters that must be changed are highlighted in pink. (TIF) [file pcbi.1004211.s007.tif]

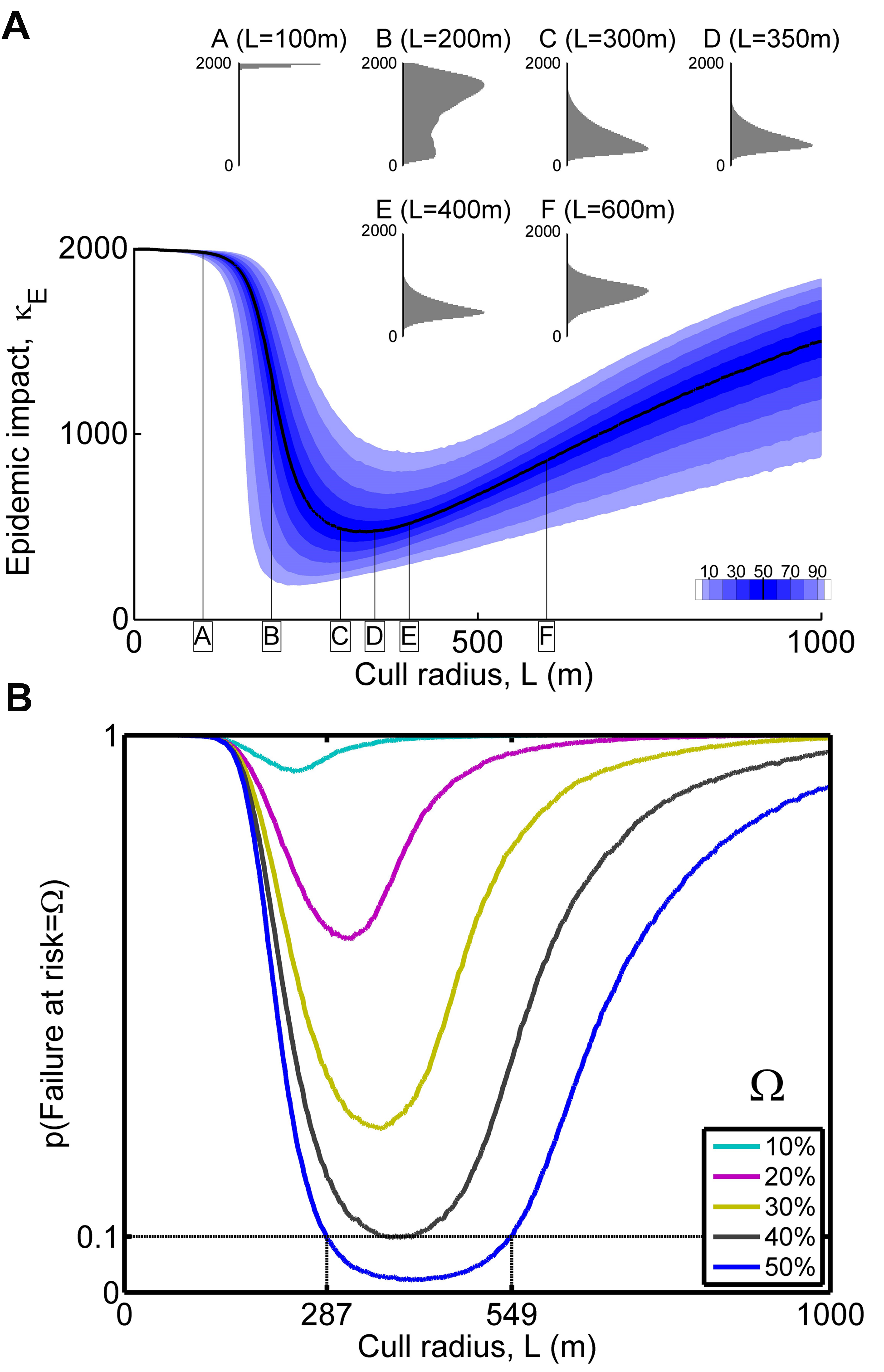

Supplement: S5 Fig — (a) Epidemic impact κE (total number of hosts lost to disease or control) as a function of the cull radius, L. This replicates Fig 2a in the main text using the parameterisation originally developed by Cook et al. [24] and used in the subsequent analyses by Parnell et al. [11,12]. (b) Risk of failure. Given a notion of “acceptable risk” (i.e. a value of Ω, the threshold κE as a percentage of the total population), the probability of failing to achieve κE < Ω is shown. Dotted line marks radii with < 10% risk of failure for Ω = 50% (range 287m < L < 549m). This is equivalent to Fig 2b in the main text but using the parameterisation originally developed by Cook et al. [24] and used in the subsequent analyses by Parnell et al. [11,12]. (TIF) [file pcbi.1004211.s008.tif]

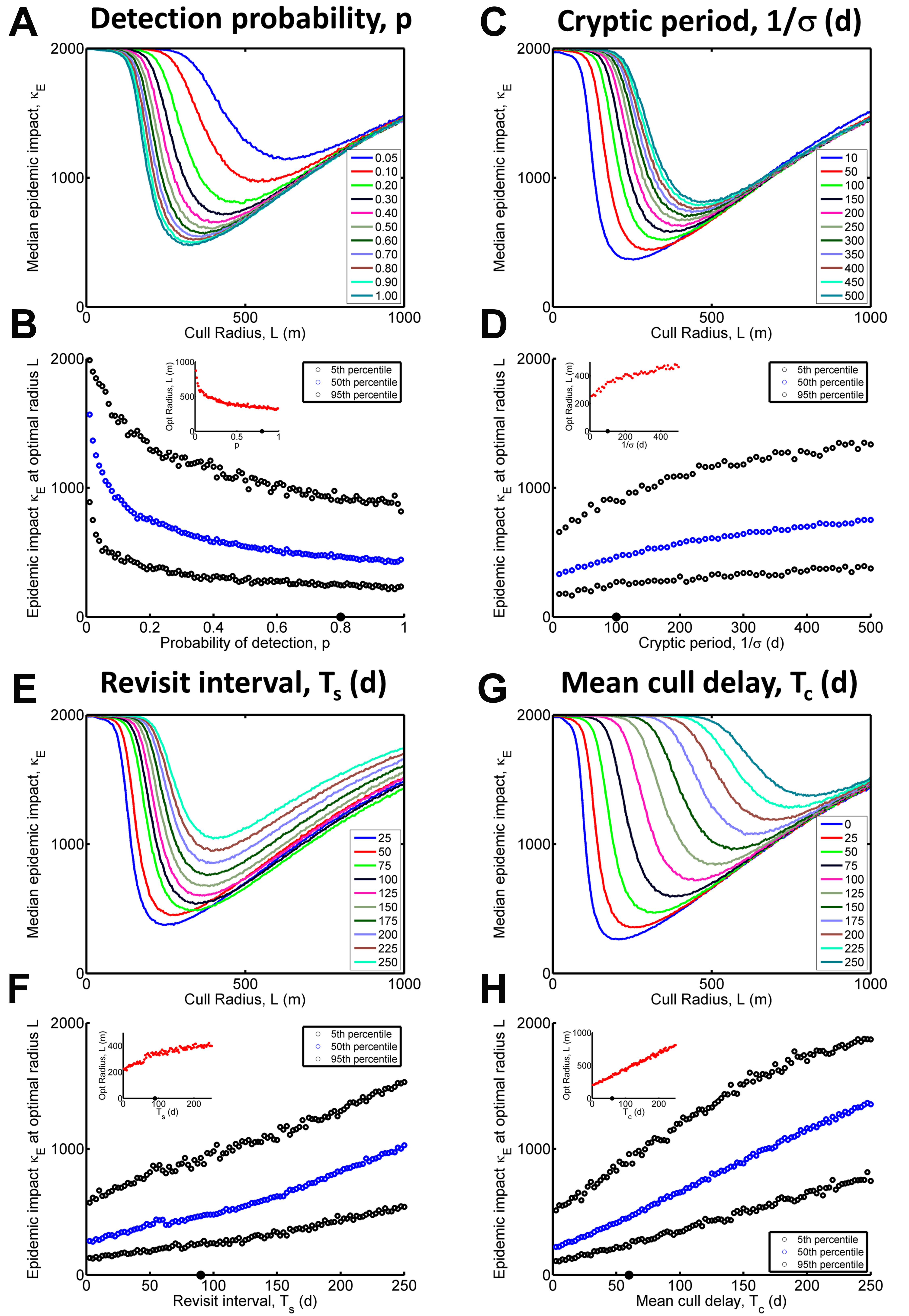

Supplement: S6 Fig — (a),(c),(e) and (g): Responses of median epidemic impact (κE) to cull radius (L) for different values of probability of detection, p (a), the average cryptic period, 1/σ (c), the interval between successive surveys, T s (e) and the notice period before culling, T c (g). (b), (d), (f) and (h): How the performance of the optimum control strategy is affected by changes in p (b), (d), T s (f) and T c (h). Insets show the response of the optimum cull radius L. The parameter values fitted by Cook et al. [24] (cf. S4 Fig) were used for all parameters except that being scanned over: these are marked with black dots on the x-axis in (b), (d), (f) and (h). This is equivalent to Fig 3 in the main text but using the parameterisation originally developed by Cook et al. [24] and used in the subsequent analyses by Parnell et al. [11,12]. (TIF) [file pcbi.1004211.s009.tif]
